# Genome engineering of Stx1-and Stx2-converting bacteriophages unveils the virulence of the dairy isolate *Escherichia coli* O174:H2 strain UC4224

**DOI:** 10.3389/fmicb.2023.1156375

**Published:** 2023-06-23

**Authors:** Giovanni Milani, Mireya Viviana Belloso Daza, Claudia Cortimiglia, Daniela Bassi, Pier Sandro Cocconcelli

**Affiliations:** Dipartimento di Scienze e Tecnologie Alimentari per una Filiera Agro-Alimentare Sostenibile (DISTAS)Università Cattolica del Sacro Cuore, Piacenza, Italy

**Keywords:** Shiga toxin-producing *Escherichia coli* (STEC), O174, Stx-converting bacteriophages, food model, raw milk cheese, *Galleria mellonella*, genome engineering, locus of adhesion and autoaggregation

## Abstract

The past decade witnessed the emergence in Shiga toxin-producing *Escherichia coli* (STEC) infections linked to the consumption of unpasteurized milk and raw milk cheese. The virulence of STEC is primarily attributed to the presence of Shiga toxin genes (*stx1* and *stx2*) carried by Stx-converting bacteriophages, along with the intimin gene *eae*. Most of the available information pertains to the “Top 7” serotypes associated with STEC infections. The objectives of this study were to characterize and investigate the pathogenicity potential of *E. coli* UC4224, a STEC O174:H2 strain isolated from semi-hard raw milk cheese and to develop surrogate strains with reduced virulence for use in food-related studies. Complete genome sequence analysis of *E. coli* UC4224 unveiled the presence of a Stx1a bacteriophage, a Stx2a bacteriophage, the Locus of Adhesion and Autoaggregation (LAA) pathogenicity island, plasmid-encoded virulence genes, and other colonization facilitators. In the *Galleria mellonella* animal model, *E. coli* UC4224 demonstrated high pathogenicity potential with an LD_50_ of 6 CFU/10 μL. Upon engineering *E. coli* UC4224 to generate single and double mutant derivatives by inactivating *stx1a* and/or *stx2a* genes, the LD_50_ increased by approximately 1 Log-dose in the single mutants and 2 Log-doses in the double mutants. However, infectivity was not completely abolished, suggesting the involvement of other virulence factors contributing to the pathogenicity of STEC O174:H2. Considering the possibility of raw milk cheese serving as a reservoir for STEC, cheesemaking model was developed to evaluate the survival of UC4224 and the adequacy of the respective mutants as reduced-virulence surrogates. All tested strains exhibited the ability to survive the curd cooking step at 48°C and multiplied (3.4 Log CFU) in cheese within the subsequent 24 h. These findings indicate that genomic engineering did not exert any unintended effect on the double *stx1*-*stx2* mutant behaviour, making it as a suitable less-virulent surrogate for conducting studies during food processing.

## 1. Introduction

Infections caused by Shiga toxin producing *Escherichia coli* (STEC) are responsible for outbreaks of serious diseases such as haemorrhagic colitis (HC) and haemolytic uremic syndrome (HUS), posing a serious public health concern (Pedersen et al., 2018). In 2020, 28 European countries reported 4,824 confirmed cases of infection with *E. coli* STEC and thus, recognized as the fourth most reported zoonosis (ECDC, 2022). Cattle have been recognized as an asymptomatic natural reservoir of STEC, representing a vehicle for human infection through direct contact or via foodstuffs (Zuppi et al., 2020). Recently, STEC outbreaks have been increasingly related to the consumption of dairy products; in Europe, two outbreaks in 2020 and one in 2021 as reported by EFSA-ECDC (EFSA and ECDC, 2021, 2022); in 2019, 20 paediatric cases of STEC O26:H11 infections were associated to the consumption of fresh raw milk cheese in France and other 21 cases were related to a milk pasteurisation malfunction at dairy farm level in UK (Jones et al., 2019; Jenkins et al., 2022). These outbreaks, as reported by the data collected in the European Union One-Health (2022) report, demonstrate as raw milk cheese and other dairy products are frequently associated to the presence of STEC (2% of analysed dairy products (EFSA and ECDC, 2022)). Thus, in the absence of an effective pasteurisation process, the cheese production and ripening steps have proven to be insufficient to achieve the complete inactivation of these pathogenic *E. coli* microorganism (Bellio et al., 2018; Ioanna et al., 2018), as shown in studies that investigated the persistence of STEC in raw milk and its derivatives (Miszczycha et al., 2013; Peng et al., 2013; Ahmed and Samer, 2017).

The current framework for identification of STEC includes the determination of serogroup, with correlation to their capacity to cause human illness. Serogroups O157, O145, O111, O103, and O26, considered the “top 5” STEC, have been identified as responsible for severe diseases and outbreaks (Franz et al., 2019; Koutsoumanis et al., 2020). Shiga toxins Stx1 and Stx2, encoded by genes *stx1* and *stx2* carried by lambdoid prophages, are considered the central driver of STEC virulence. Each of the Stx toxins are furtherly classified into subtypes and, particularly Stx2 subtypes a and c, seem to be corelated to the most severe forms of STEC diseases (Werber and Scheutz, 2019; Rodríguez-Rubio et al., 2021). The risk for a severe disease is generally associated with the concurrent presence of the *stx2* gene and the Locus of Enterocyte Effacement (LEE), which contains the *eae* gene, coding for the intimin protein responsible for adhesion (Franzin and Sircili, 2015). However, recently, non-O157 LEE-negative strains have been correlated with increasing number of infections in humans (Cundon et al., 2018; Krause et al., 2018; Colello et al., 2019; Cortimiglia et al., 2021). The LEE-negative STEC strains implicated in human disease harbour other virulence factors (VFs) involved in other adherence processes carried by plasmids, non-Stx prophages or unique pathogenicity islands (PAIs). Montero et al. (Montero et al., 2017), described the PAI Locus of Adhesion and Autoaggregation (LAA), a 86 kb region divided in four modules containing the *hes* gene coding for and haemagglutinin (Montero et al., 2017). A recent study by Cortimiglia et al. (Cortimiglia et al., 2021) detected this virulence locus in STEC O174 strains harboring both Stx1- and Stx2-bacteriophages isolated from Italian semi-hard raw milk cheese. Moreover, *E. coli* O174 strains are frequently detected as being among the top 10 STEC serotypes from animal, food and humans (EFSA and ECDC, 2022).

Although the risk that STEC poses for consumers of dairy products is high, few studies have addressed the growth, survival and inactivation kinetics of Shiga toxin producing *E. coli* during the cheese processing and ripening (Schlesser et al., 2006; Miszczycha et al., 2016; Centorotola et al., 2021). One of the major limitations in the development of challenge studies in food, is the high pathogenicity of STEC strains that hamper their use in pilot plants outside the confined conditions of biosafety laboratories. STEC mutants with the toxin genes inactivated were developed to assess the role of Stx virulence (Kim et al., 2010; Xue et al., 2011) but not specifically used as surrogate for toxigenic strains to appraise the growth and persistence in food models. The objective of our study is to perform a comprehensive genomic characterization of the virulence profile of *E. coli* UC4224, a STEC strain isolated from semi-hard raw milk cheese, utilizing a WGS-based approach. Our research also aims to investigate the impact of *stx1* and *stx2* genes on pathogenicity *in vivo* using the *Galleria mellonella* model by individually and collectively inactivating them via genome engineering. Moreover, we assessed the survival of the parental strain and the suitability of the three mutants as attenuated surrogates under acid stresses and in cheesemaking conditions.

## 2. Methods and materials

### 2.1. Bacterial strains, plasmids, and media

STEC strain UC4224, isolated from semi-hard raw milk cheese, and respective mutant strains were grown in Luria-Bertani (LB) broth (Sigma-Aldrich) and supplemented with appropriate antibiotics when needed. The antibiotics used were chloramphenicol (Cm) (Sigma-Aldrich) (3.125–25 μg/mL), kanamycin (Kan) (Sigma-Aldrich) (12.5–25 μg/mL), and ampicillin (Amp) (Sigma-Aldrich) (100 μg/mL). Strains and plasmids used in this study are listed in Table 1 whereas oligonucleotides are listed in Supplementary Table S1. The *E. coli* strain DH5α, grown in LB broth supplemented with Amp, was used for the propagation and purification of plasmids.

**Table 1:** Bacterial strains and plasmids used in this study.

Strain	Relevant genotype, phenotype	Reference/Source
*E. coli*		
UC4224	STEC parental strain	This study
UC4175	UC4224(pSIM6), Amp^R^ (Ts)	This study
UC4176	UC4224*Δstx1::kan*, Kan^R^	This study
UC4177	UC4224*Δstx2::cat*, Cm^R^	This study
UC4178	UC4224*Δstx1::kan Δstx2::cat*, Kan^R^ Cm^R^	This study
*Plasmids*		
pSIM6	Plasmid expressing Lambda red recombination genes below the control of CI857 repressor, Amp^R^ (Ts)	Datta et al. (2006)
pKD3	Template plasmid for the amplification of FRT-cat-FRT amplicon, Amp^R^ Cm^R^	Datsenko and Wanner (2000)
pRL128	Template plasmid for the amplification of FRT-kan-FRT amplicon, Amp^R^ Kan^R^	Gueguen and Cascales (2013)

Amp, ampicillin; Kan, kanamycin; Cm, chloramphenicol; superscripts “R” and “S” represent resistance and sensitivity, respectively.

### 2.2. Whole genome sequencing and data submission

Genomic DNA of UC4224 and UC4178 (UC4224*Δstx1::kan Δstx2::cat*) was extracted from 1 mL of an overnight culture by E.Z.N.A. ® Bacterial DNA Kit (Omega Bio-tek), following the manufacturer’s instructions. After, Qubit 2.0 Fluorometer (Thermo Fisher Scientific) was utilized to quantify the DNA concentration and then loaded on agarose gel (0.8%) to confirm the DNA integrity. Genomic DNA of UC4224 and UC4178 were sequenced using Illumina Miseq platform with 250 paired-end run after Nextera XT paired-end library preparation (Illumina). Additionally, long-read sequencing was carried out for UC4224 only and performed with PacBio Sequel II SMRT sequencing. Sequence trimming was completed with trimgalore! (GitHub – FelixKrueger/TrimGalore) (Krueger, 2016). After, hybrid assembly was executed using Unicycler (Wick et al., 2017). Then, contigs of both parental and mutant strains, were annotated with Prokka with a de fault e-value cut-off (version 1.13.3) (Seemann, 2014). Genome assemblies were deposited on NCBI under Genbank assembly accession No. GCA_025369975.1 for UC4224 and GCA_025290845.1 for UC4178.

### 2.3. Bioinformatic analyses

A total of 99 strains genomes (including UC4224) were retrieved from NCBI for phylogenomic analyses, including 40 from cheese, 5 from dairy milk and 54 from non-specified dairy products (Supplementary Table S2). Bioinformatic analyses comprising the calculation of the pangenome and the construction of the phylogenetic tree with bootstrapping of 1,000, of all genomes were performed as previously described by Belloso Daza et al. (2021). The screening for virulence factors, antimicrobial resistance genes was executed according to another study (Cortimiglia et al., 2021). Finally, the analysis of mobile genetic elements like plasmids and prophages was carried out following the pipeline of Belloso Daza et al. (2022).

### 2.4. Construction the amplimer with short (50 bp) and long (~280 bp) homology sequences

PCR reactions were performed using Phusion Flash High-Fidelity PCR Master Mix (ThermoFisher Scientific) as provided by the manufacturer. The two plasmids pKD3 (Datsenko and Wanner, 2000) and pRL128 (Gueguen and Cascales, 2013) were used for amplifying the resistance cassettes using primers (Supplementary Table S1) constructed as described by Egan et al. (2016). The detection of each amplicon was verified by gel electrophoresis (ThermoFisher Scientific), then the product was excised from the gel and purified using the Macherey-Nagel™ NucleoSpin™ Gel and PCR Clean-up (Macherey-Nagel). The PCR product was concentrated using Zymo Research’s DNA Clean & Concentrator Kit™-25 (D4005) in a final volume of 25 μL of molecular-grade water.

Homology regions of the *stx2* gene, located at the two ends of the *cat* cassette, have been increased as previously described by Serra-Moreno et al. (2006). The new PCR products were constructed using the overlapping regions within three different dsPCR fragments: the *cat* cassette and the other two that present homology with both *stx2* and antibiotic resistance cassette using the primers reported in Supplementary Table S1. The amplimers obtained were *stx*2 Forward/Cm-F *stx2* (270 bp) and *stx*2 Reverse/Cm-R *stx2* (280 bp). The three amplimers obtained were annealed at their overlapping region. The two external primers *stx*2 Forward and *stx*2 Reverse were used to overlap the three fragments. The fusion product was amplified using the same primer pair *stx*2 Forward/Reverse, subsequently purified. The fusion product obtained is *Δstx2::cat* PCR amplicon with long homologous arms.

### 2.5. Transformation of *Escherichia coli* STEC UC4224 with plasmids pSIM6 and preparation of electrocompetent cells for recombineering

The pSIM6 plasmid was propagated in *E. coli* strain DH5α and extracted using ZymoPURE Plasmid Miniprep Kit (Zymo Research) following the manufacturer instructions. Then it was transformed in UC4224 after making it electrocompetent (BIORAD, 1900) *E. coli*. The transformant of UC4224 with the pSIM6 was named UC4175. The UC4175 overnight culture was then diluted to 100-fold in LB with Amp (100 μg/mL) and grown to an OD_600_ of 0.8. The culture was then thermally shocked at 42°C at 250 rpm for 45 min to induce the lambda red genes expression by pSIM6, as described previously (Egan et al., 2016). After the induction, UC4175 was made electrocompetent as described above. Ninety μl of chilled electrocompetent UC4175 cells were added to 100 ng of *Δstx2::cat* or *Δstx1::kan* PCR amplicons, including negative controls without PCR products. The mix was held on ice for 1 min, then, electroporation was performed at a voltage of 2.5 kV. Electroporants were immediately recovered in 1 mL of S.O.C medium and grown at 37°C at 225 rpm overnight,. After, the cultures were spread on LB supplemented with Cm (6.5–25 μg/mL) or Kan (15–30 μg/mL) and examined to determine Cm^R^ and Kan^R^ recombinants. Recombinants were observed after 1 to 2 days of incubation at 37°C. The resulting mutants are UC4176 (UC4224*Δstx1::kan*, Kan^R^) and UC4177 (UC4224*Δstx2::cat*, Cm^R^) (Table 1).

Subsequently, UC4176 was induced and made eletrocompetent, as described above. The induced eletrocompetent cells were eletroporated with 100 ng of *Δstx2::cat* PCR amplicons with long homologous arms. The electroporation conditions used were the same as those described previously. After 16 h of incubation, recovered cultures were cultured onto LB plates with Cm (6.5–25 μg/mL) and Kan (15–30 μg/mL) and examined to determine Cm^R^/Kan^R^ recombinants. The resulting double mutant is UC4178 (UC4224*Δstx1::kan Δstx2::cat, Kan^R^ Cm^R^*) (Table 1).

### 2.6. Curing of pSIM6 and replacement confirmation

Once the recombinant UC4178 had been found, 10 μL of an overnight culture was spread on LB agar with added Cm (6.25 μg/mL) and Kan (22.5 μg/mL) and incubated overnight at 42°C. A few colonies were then taken and streaked onto both LB agar supplemented with respective antibiotics and incubated overnight at 30°C. The correct replacement was confirmed by locus specific PCR and Sanger sequencing. Briefly, the *stx1* and *stx2* genes were amplified with the external primer listed in Supplementary Table S1 (Paton et al., 1993, 1995; Ruessmann et al., 1994; Muniesa et al., 2003), visualized on 1.2% agarose gel by Sybr-Safe staining (Thermofisher) and purified using ReliaPrep DNA clean-up and concentration system (Promega) according to the protocol provided by the manufacturer. The purified DNA was sequenced by commercial facility (Eurofins Genomics, Italy) using Sanger technology. Additionally, the replacement was confirmed through WGS, performed as reported above. The genome assembly is deposited in Genbank with accession number GCA_025290845.1.

### 2.7. Pathogenicity assessment in *Galleria mellonella*

The *in vivo* analysis using larvae of the greater wax moth, *G. mellonella*, was performed as previously described by Morgan et al. (2014). Briefly, bacterial overnight cultures were pelleted and washed twice in Phosphate Buffer Solution (PBS) (0.1 M) and resuspended in 10 mL of PBS. The larvae were selected to be 15–25 mm long, cream-coloured with minimal spotting and no grey marks. Three independent biological replicates of ten larvae of 200 to 250 mg each, were infected with 10 μL of serial dilution (10^1^–10^7^ CFU/10 μL) of each different strains, by injection with a 26-gauge Hamilton syringe. Larvae were then incubated at 37° C in the dark and the dose resulting in 50% of kills (LD_50_) was calculated after 24 h. The survival rate was monitored for an additional 48 h. The strains used for this assay were UC4224, UC4176, UC4177, UC4178 and *E. coli* BL21 and PBS only as a negative control. An additional control composed by three groups (*n* = 10) without manipulation, was added. Kaplan–Meier survival curves were constructed to evaluate the probability of survival of the different strains at different injection doses using GraphPad Prism (Survival curve 8.4.3 (686)). Logrank tests were applied to detect any significant differences in survival rates between strains (*p < 0.05*). Microbial count of bacteria was realized to verify the inoculated doses onto LB agar supplemented with Kan and Cm, when needed. The LD_50_ values were calculated using Probit Analysis, following the methodology of Finney (1971) in Excel 2010 with a 95% confidence limit (Mekapogu, 2021).

### 2.8. Cheesemaking model and tolerance to lactic acid

We assessed the survival capacity of UC4224 and the suitability of UC4176, UC4177 and UC4178 as attenuated surrogates under acid stresses and in cheesemaking process. The cheesemaking process was carried out according to the traditional production method from raw milk. Briefly, 200 mL of raw milk was aliquoted in five different 500 mL flasks and pre-warmed at 30°C. Once the desired temperature was reached, each flask was inoculated with a mix of three different starter cultures: *Streptococcus thermophilus*, *Lactococcus lactis* and *Lactobacillus delbrueckii* subsp. *lactis* at a cell numbers of 10^7^ CFU/m each, and 0.2 mL of rennet per litre of milk. Subsequently, four flasks prepared as described above were individually inoculated with 200 μL of an overnight culture of UC4224 and the three mutants (inocula-t0); the remaining flask, without *E. coli* inoculum, was used as a negative control. The five samples were heat-treated at 34°C for 40 min (t1); then, the temperature was increased at 48°C for 40 min (t2) and finally the curds were packaged, pressurised and drained; thus, stored at room temperature (20°C) for 24 h (t3). Plate counts were carried out in triplicate at times t0, t1, t2 and t3 using Violet Red Bile Glucose Agar (Oxoid), supplemented with Kan 50 μg/mL and Cm 50 μg/mL when required, and incubated at 37°C for 24 h. Lactic acid tolerance was tested as previously described by Liu et al. (2020) with slight variations. Shortly, the overnight culture of the parental strain and the three mutants were serially diluted and 10 μL of each dilution were spotted on LB agar, modified with L-lactic acid (Carlo Erba) to a pH of 4, 4.5, 5, 5.5, 6 and 6.5, and incubated at 37°C for 24 h. All results were statistically analysed using the Tukey’s pairwise test, via the *Past4.06b* software, with *α* = 0.05.

## 3. Results

### 3.1. Genome sequencing and characterization of UC4224

In this study, *Escherichia coli* STEC strain UC4224, isolated from semi-hard raw milk cheese, was investigated for its resistome/virulome/mobilome. The first step toward identifying the nature of this strain was to perform WGS following a long-and short-read approach. After sequencing and quality check, UC4224 was assembled into 3 molecules, one chromosome of 5,047,333 bp and two plasmids, pUC4224_1 (111,840 bp) and pUC4224_2 (6,883 bp). UC4224 was identified as ST 661, serotype O174:H2 and Clermont phylogroup B1.

### 3.2. Phylogenomics and distribution of dairy associated STEC

**Figure 1:**
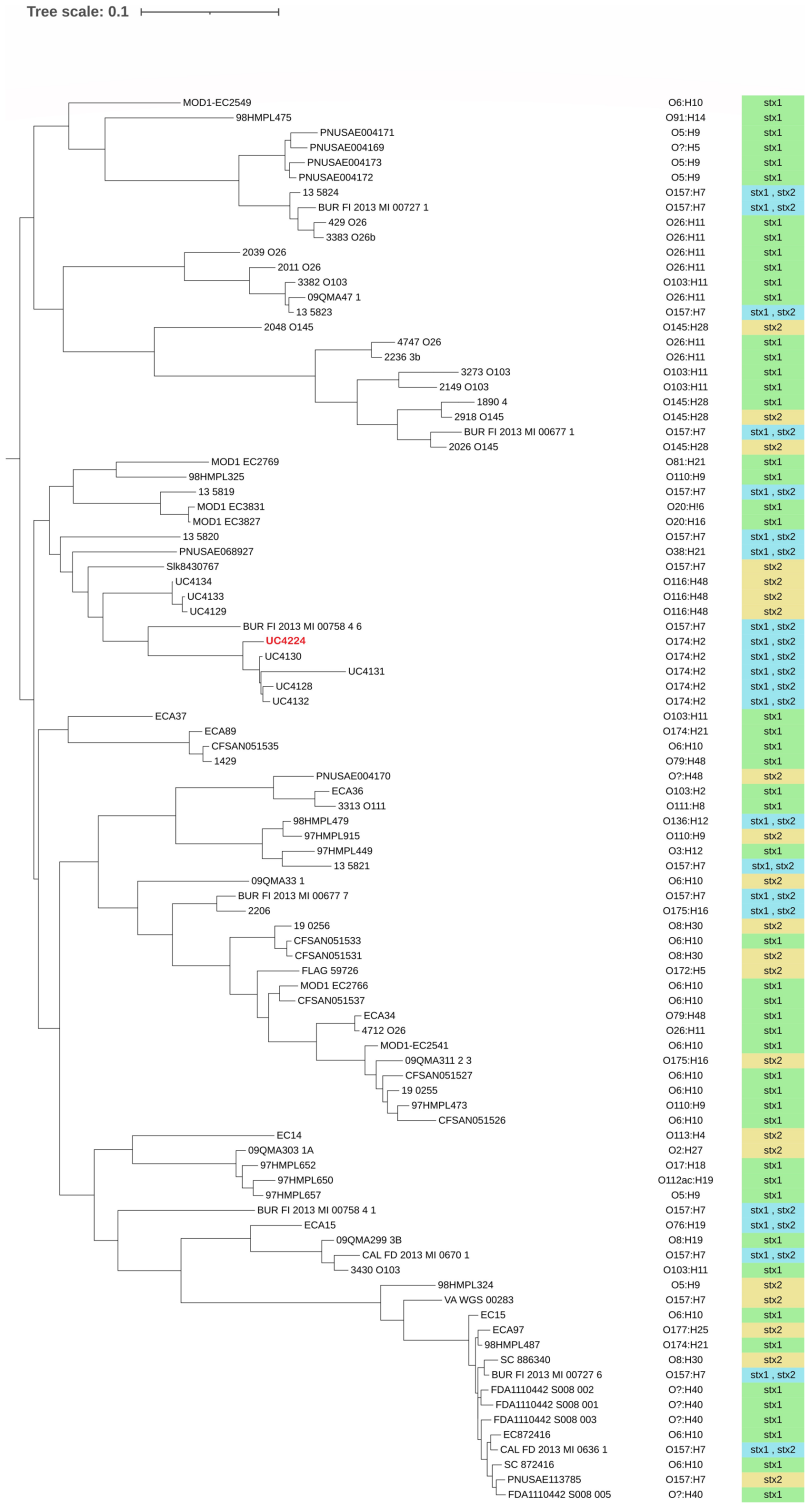
Maximum likelihood phylogenetic tree using core genes alignment of 99 dairy-associated strains retrieved from NCBI. UC4224 is depicted in red. The serogroup of each isolate was determined using a bioinformatic tool and it is presented on the right side of the three. The type of Shiga toxin is depicted in green for Stx1, yellow for Stx2 and blue for Stx1 and Stx2. The variability of the presence of Stx-type is correlated to the serogroup. No clear patterns in serogroup or Stx-type distribution is observed. The selected strains had different isolation sources: milk, cheese and dairy products; their distribution can be found in Supplementary Table S2.

Phylogenomic analysis was performed to understand the relationship of *E. coli* UC4224 with a selection of 95 *E. coli* genomes, retrieved from NCBI, isolated from dairy-associated sources: milk, cheese and dairy products. The pangenome analysis resulted in a total of 2,175 (8.7%) core genes, 2,652 (8.3%) shell genes and 25,653 (83%) cloud or accessory genes. This outcome is in line with the concept of the open pangenome of *E. coli*, decreasing core genomes and increasing accessory genomes that support the adaptability of *E. coli* from different ecological niches and the diversity of strains with pathogenicity for animals and human (Tantoso et al., 2022). Furthermore, core-genome derived data was then used to construct a maximum likelihood phylogenetic tree. As observed in Figure 1, no clear cluster patterns regarding serogroup or Stx type are noticeable. When observing the relative abundance of the serogroups we found the most frequent serogroups were O157 (17%), O6 (13.8%), O26 (8.5%) and O174 (7.4%). Other recurring serogroups were O103 (6.4%), O5 (5.3%) O8 (4.3%), O110 (3.2%), O116 (3.2%), O145 (4.3%). Out of 94, only 6 isolates (6.4%) were not assigned to any serogroup. A strict relationship between the Stx-bacteriophages and serogroups results from the phylogenomic analysis, as reported in other studies (Zhang et al., 2022). In particular, O174 strains harbour both Stx1- and Stx2-converting phages and O6 and O26 dairy isolates carry Stx1. Higher variability was observed in O157 which may contain either Stx2 – or both Stx1- and Stx2-phages.

### 3.3. Stx-converting phages and other prophages

**Figure 2:**
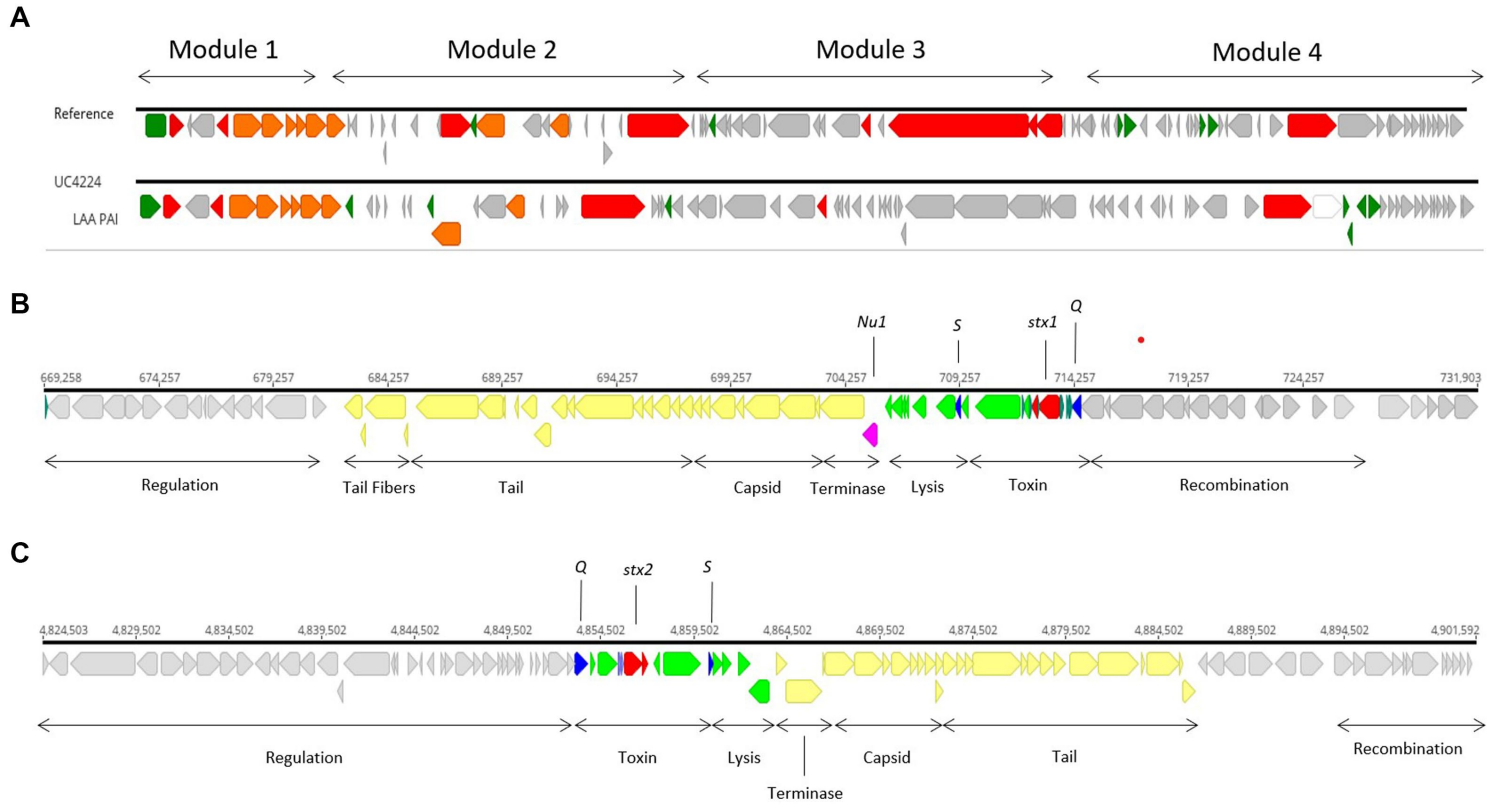
Stx1- and Stx2-converting phages and LAA PAI in UC4224. **(A)** sequence alignment of LAA PAI reference sequence from *E. coli* B2F1 (Genbank accession AFDQ01000026) and UC4224. **(B)** genomic annotation of Stx1-phage including stx1a toxin subunits (red), antiterminator protein Q and holin S (blue), Nu1 protein (magenta), lysis and toxin operon CDS (green), structural proteins for capsid and tail (yellow) and, regulation, recombination and other CDS (grey). Stx 1 attachment site attL was found in position 669,258–669,271 bp (13 bp, TGCCGGATGCGGCG) and attR in position 731,916–731,903 (13 bp, TGCCGGATGCGGCG). **(C)** genomic annotation of Stx2-phage including stx2a toxin subunits (red), antiterminator protein Q and holin S (blue), lysis and toxin operon CDS (green), structural proteins for capsid and tail (yellow) and, regulation, recombination and other CDS (grey). Stx2-phage presented attL in position 4,830,701–4,830,714 bp (13 bp, TGGATGATTTTTCA) and attR in 4,901,592–4,901,603 bp (11 bp, TTATGAAAAACG).

Genome scrutiny of UC4224 revealed the presence of *stx1a* and *stx2a* carried by two separate prophages. We comprehensively explored the two Stx-phages and their respective flanking regions by identifying the attachment sites, structural and regulatory regions. Stx-phages are double-stranded DNA-phages with a functional genetic organization comparable lambda phage, as it is the case of UC4224 phages. Stx1-phage of size 62.2 kb (Figure 2B), was found in positions 669,258–731,903 bp, with the highest homology score to Enterobacteria phage D3 (NC_042057). Stx2-phage, of size 77 kb (Figure 2C), found in position 4,824,523–4,901,603 bp with highest similarity to phage D3, as well. Both phages were found to be unique with the highest BLAST nucleotide identity of 92 and 88% with other Stx-phages for Stx1-phage and Stx2-phage, respectively.

The *stx* genes loci of both phages, were composed by the two *stx* genes coding for subunits a and b, the antitermination protein Q, responsible for late-phase transcription regulator and lysis protein S. Downstream from the Stx-region of both phages, lysis, terminase and structural proteins coding for capsid, tail and tail fibers were observed. Regulation and recombination genes were found adjacent to the toxin and structural genes regions. Upstream from the Stx-region, recombination coding sequences were found (Figures 2B,C). Specifically, in Stx1-phage, gene *nu1* was found, coding for a typical protein for DNA packaging in Stx-converting phages (Figure 2B). Stx2-phage carries *perC,* a Type 3 Secretion System (T3SS) expression regulator related to the expression of LEE-encoded virulence factors in STEC (Carter et al., 2021).

Moreover, four additional prophages were predicted chromosomally, namely a 25.4 kb phage with highest homology to Enterobacteria phage YYZ_2008 (Acc. No. NC_011356), two phages of 39.1 kb and 44.7 kb similar to Enterobacteria phage lambda (Acc. No. NC_001416) and a 46.9 kb closest to *Klebsiella* phage 4 LV-2017 (Acc. No. NC_047818). The 44.7 kb phage carried gene *ompT* (outer membrane protein and serum resistance lipoprotein *bor* was found flaking the lysis and terminase regions of this phage. Downstream from the structural region and adjacent to the tail fibers coding genes, *lom* (outermebrane protein) was found (data not shown).

### 3.4. Virulence factors and LAA pathogenicity island

Although *stx* genes are considered the main drivers of virulence, *E. coli* STEC strains have developed pathogenicity islands (PAI) carrying genes for adhesion and colonization and attachment that facilitate the expression of virulence within the host. *E. coli* UC4224 does not harbour the Locus of Enterocyte Effacement (LEE) PAI, a 35.6 kb region containing genes responsible for causing attaching and effacing lesions, characteristic of *E. coli* O157: H7 (Franzin and Sircili, 2015). Differently, the WGS scrutiny revealed the presence of a region showing 60.8% nucleotide similarity with the Locus of Adhesion and Autoaggregation (LAA) PAI (Genbank Acc No. AFDQ01000026) (Figure 2C), a genetic locus described by Montero et al. (Montero et al., 2017). As shown in Figure 2A, module 1 carries the gene *hes*, involved in self-aggregation and adhesion (Vélez et al., 2020a). Module 2 habours the *lesP* gene, which encodes a variant of an enterobacterial self-transporting serine protease (SPATE) (Montero et al., 2019). Module 3 has the *pagC,* an outer membrane protein important in serum resistance in *Salmonella enterica* (Hasson et al., 2022). Finally, the *agp43* gene is found in module 4, which is related to the capacity for self-aggregation and accumulation of cells, which promotes biofilm formation (Montero et al., 2017).

Other two PAIs have been described to appear in LEE-negative STEC strains, specifically: the Locus of Proteolysis Activity (LPA) (Hauser et al., 2013) and the Subtilase-Encoding Pathogenicity Island (SE-PAI) (Bondì et al., 2017) were not found in *E. coli* UC4224. Other chromosomally located genes encoding for adhesins, T3SS effectors and potential virulence factors were identified, including *hra* (heat-resistant agglutinin) and long polar fimbriae (*lpfA*), an important factor for STEC intestinal colonization and adhesion (Supplementary Table S3) (Toma et al., 2006; Vélez et al., 2022).

The IncF-type conjugative plasmid pUC4224_1 (111 kb), carries a large Integrative Conjugative Element (ICE) in position 25,634–106,706 bp (81,073 bp). This ICE presents an origin of transfer (*oriT*), Type 4 Secretion System (T4SS) proteins tra and trb and Type IV coupling protein (T4CP) in ORF 48 (795 aa). Moreover, pUC4224_1 carried several potential virulence factors (Supplementary Table S3), among them adherence protein *iha,* enterohemolysin operon *ehxABCD*. Next, gene *espP* was also found, these genes are homologues members of Serine Protease Autotransporters of Enterobactericeae (SPATE) family. The *traT* gene, a plasmid-located determinant encoding for an outer membrane protein that inhibits the membrane-attack complex present in the serum of the host (Miajlovic and Smith, 2014) and *saa* (STEC autoagglutinating adhesin) genes (Cundon et al., 2018) were found as potentially involved in virulence. This strain harbours colicin coding genes *cia* and *celb*, considered as a putative virulence factors as they facilitate colonization (Micenková et al., 2017). Furthermore, several stress response systems regulators were found in UC4224, gene list with corresponding gene function are listed in Supplementary Table S3.

### 3.5. Construction of *stx1* and *stx2* null mutants

**Figure 3:**
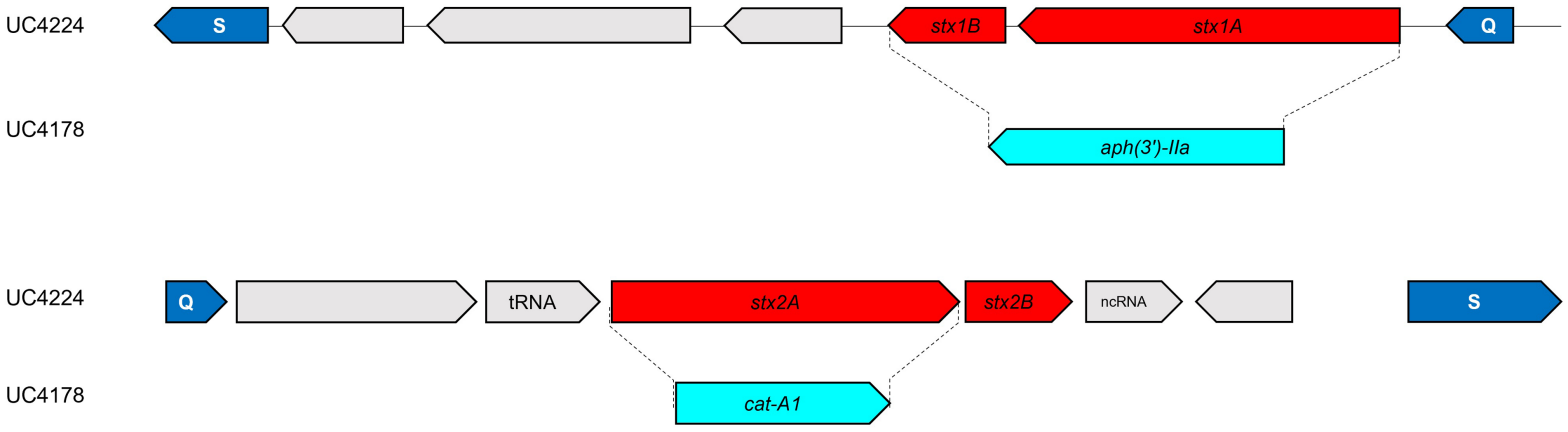
Schematic representation of stx1 and stx2 replacement in UC4224. Genes are represented by arrows. In cyan the antimicrobial resistance cassettes. In red the two subunits, respectively, of stx1 and stx2 genes. In the double mutant UC4178, the replacement event occurred via homologous recombination between stx1 subunits a/b and the kanamycin resistance cassette and between stx2a and the chloramphenicol resistance cassette. The dimension of the amplified stx1 and stx2 genes, with the external primers, in the parental strain UC4224 have a size, respectively, of 1,281 bp and 1,241 bp. Instead, the size of the same region, amplified with the same primers, in strain UC4178 are, respectively, of 1,403 bp and 1,568 bp; thus, confirming the correct gene substitution.

To investigate the role of phage encoded *stx* genes from newly identified Stx-phages from a non-O157 strain isolated from semi-hard raw milk cheese. Therefore, we constructed *stx1-* and *stx2*-knock-out strains by inserting antimicrobial cassettes by using the lambda red recombination system expressed by the low copy plasmid pSIM6, as shown in Figure 3. After the deletion of *stx* genes, PCR experiments and Sanger sequencing confirmed the substitution of the *stx1* region with the Kan^R^ (*aph(3′)-IIa*) (UC4176 and UC4178) and the *stx2a* with the Cm^R^ (*catA1*) (UC4177 and UC4178), in all the three obtained mutants as shown in Figure 3. Moreover, the WGS analyses of UC4178 confirmed the double substitution of the *stx* genes and the absence of the pSIM6 plasmid and, no other differences were observed when compared with the parental strain UC4224.

### 3.6. Lethality in the *Galleria mellonella* model of UC4224 and its derivative mutants is correlated with carriage of *stx* genes

**Figure 4:**
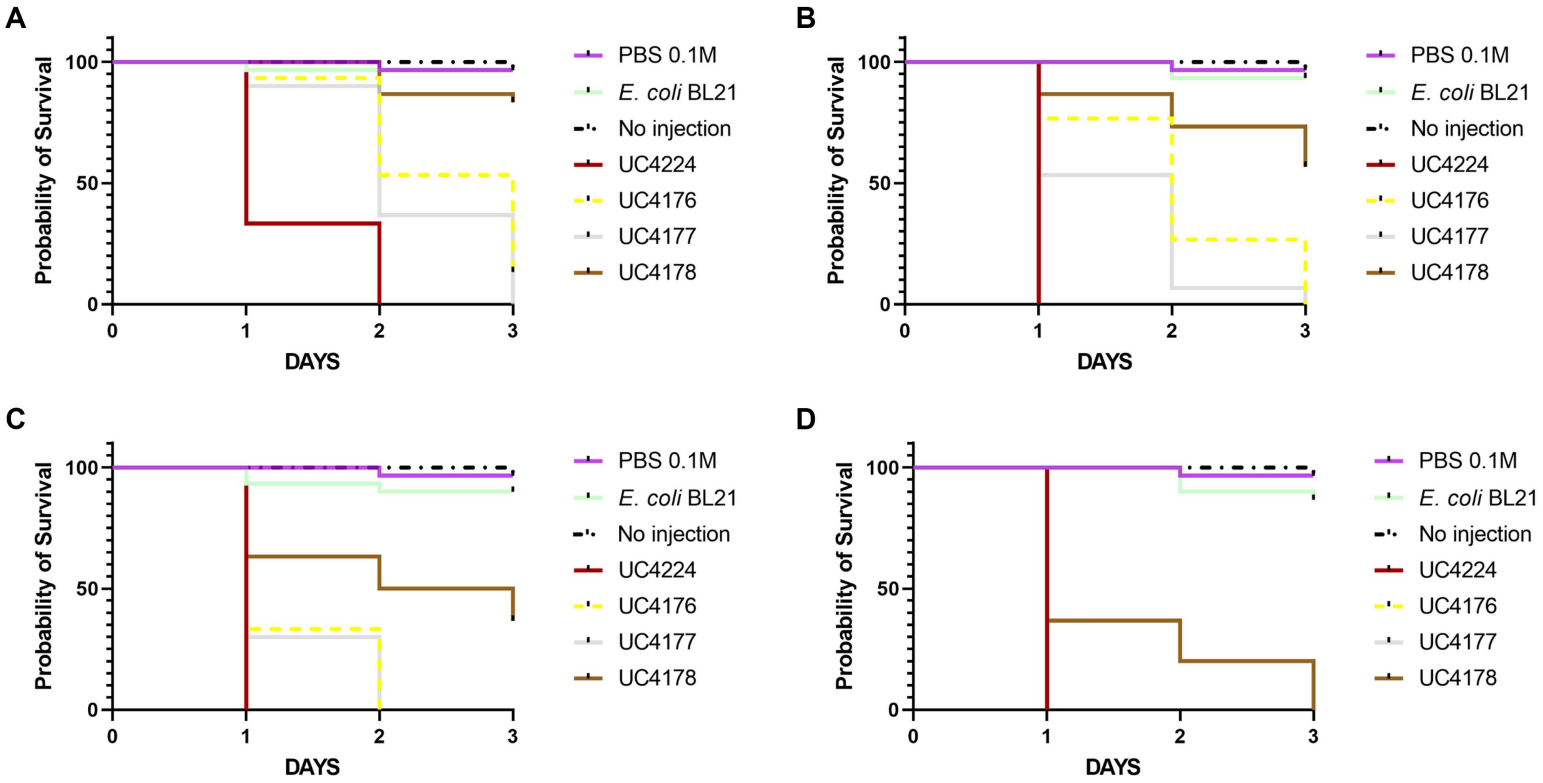
Kaplan–Meier survival curves of the experiments with G. mellonella larvae inoculated with tested strains at different injection doses **(A)** 9 CFU/10 μL, **(B)** 1.8×10^1^ CFU/10 μL, **(C)** 1.8×10^2^ CFU/10 μL, and **(D)** 1.8×10^3^ CFU/10 μL. Each group contained 30 larvae separated in three groups of 10 larvae. *E. coli*.

In this study we tested the virulence of STEC UC4224 and STEC-negative mutants UC4176, UC4177 and UC4178. To determine the mortality rates, the *G. mellonella* larvae were injected with a range of 10^1^ to 10^7^ CFU/10 μL of the mutant strains in comparison with UC4224 and *E. coli* BL21 as negative control. Larvae injected with negative controls showed no mortality. The Kaplan–Meier survival analysis (Figure 4) was based on the four lowest doses, as at 1.8×10^4^ CFU/10 μL or higher, and the observed mortality rate was 100% for all the tested strains. The parental strain UC4224, which harbours the two intact *stx* operons, has a LD_50_ of 6.0 CFU/10 μL (Supplementary Table S5.2). When the two single mutants UC4176 and UC4177 were tested, the observed mortality rates were significantly lower than UC4224 (*p* < 0.05) for the three lowest doses injected, with a LD_50_ of 81.7 CFU/10 μL and 50.5 CFU/10 μL respectively, but not significantly different between them (Supplementary Table S5.1). The lethality rate of UC4178 strain, with a LD_50_ of 582.7 CFU/10 μL, was significantly lower than the parental strains and the two single mutants for the four tested doses. *In vivo* trials with *G. mellonella* indicated an improved survival rates in larvae samples treated with the three mutants compared to those treated with the parental strain, with particular attention to UC4178 in which the deletion of the *stx1* and *stx2* genes allowed a considerable reduction in pathogenicity.

### 3.7. Survival and growth dynamics of UC4224 and derivate mutants during food processing

**Figure 5:**
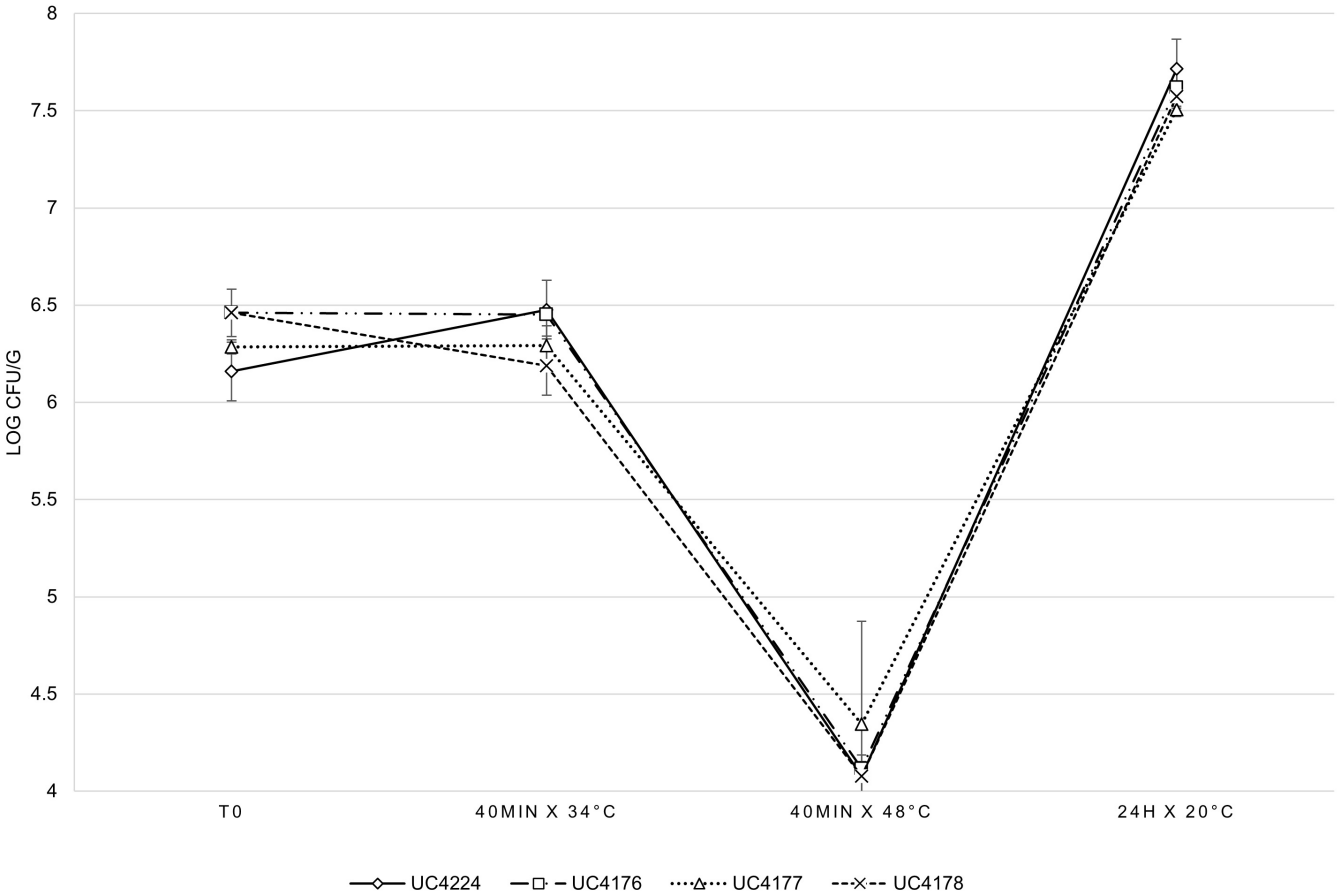
*E. coli* counts in cheese making, at different analysis times, for the four independent experimental tests expressed as the average of three independent experiments. Error bars indicate standard deviation.

A cheesemaking model, mimicking the first step of raw milk cheese production, was developed to evaluate the survival of UC4224 and the adequacy of the respective mutants as reduced-virulence surrogates during the cheesemaking process. The results of the bacterial counts, expressed as the average of three experiments, are shown in Figure 5. The four considered strains showed the same inactivation and growth dynamics in all the analysed steps of the food processing model, without statistically significant differences. In the first 40 min at 34°C, corresponding to the renneting step, no growth was observed. The thermal treatment at 48°C for 40 min, which represents the typical step of semi-hard raw milk cheese, resulted in a reduction greater than a 2 Log CFU/g for all four strains. In the subsequent step, when the curd was separated from whey and maintained at 20°C for 24 h, growth was observed reaching values of 1.3 Log and 3.4 Log to the thermal treatment at 48°C step. *E. coli* UC4224 and its Stx-phage-inactivated strains derivative were tested for their resistance to pH values typical of dairy products, obtained by adding lactic acid to growth medium. At pH 4, no growth was detected for any of the four strains at any cell density tested, at pH 4.5 growth was observed only with an inoculum concentration higher than 7 Log CFU/ml, while at pH of 5 was not growth limitation was seen (Supplementary Table S4).

## 4. Discussion

Recently, Shiga-toxin producing *Escherichia coli* (STEC) infections have been associated with the consumption of raw milk and derivatives thereof. In this study, STEC strain UC4224 was isolated from semi-hard raw milk cheese and was subjected WGS to investigate its virulence profile. Bioinformatic analyses using genome-derived data, allowed the classification of UC4224 as ST 661, serotype O174:H2 and, carrying two new and separate Stx1 and Stx2-converting phages with the typical Stx-converting phage structure (Figure 2). Many *stx*-carrying strains harbour LEE PAI but this strain was determined LEE-negative. LEE-negative strains have developed further mechanisms that facilitate infection. Commonly, LEE-negative STEC strains carry other adhesion and colonization-contributing factors like *iha* (IrgA homologue adhesin), *saa* (STEC autoagglutinating adhesin), and *lpfA* (long polar fimbria), that compensate for the absence of LEE, as it is the case of UC4224 (Lorenz et al., 2016). UC4224 harbours also LAA PAI (86 kb) in the chromosome and carries virulence factors throughout its 4 modules as previously described (Montero et al., 2017). A 44.7 kb non*-stx* prophage was found in the chromosome, with the gene *ompT*, a gene coding for a membrane protease highly associated with adhesion and pathogenicity in urinary tract infections (He et al., 2015); *lom* and *bor* genes were also found, which are involved in T3SS expression that confer serum resistance and enhance adhesion (Rodríguez-Rubio et al., 2021). It has been demonstrated that other non*-stx* prophages have a direct impact on the STEC pathogenicity and pangenome, but the direct impact on UC4224 virulence is still to be determined (Rodríguez-Rubio et al., 2021). Other plasmid encoded virulence genes were found in pUC4224_1 (111 kb), including enterohemolysin gene *ehxA*, demonstrated to contribute to virulence in STEC (Lorenz et al., 2016; Hua et al., 2021); SPATE family gene (*espP*) and other adhesion (*traT* and *saa*) genes.

Insights into the virulence profile of UC4224 led to the construction of single and double *stx1* and *stx2* knock-out mutants to study its pathogenicity potential *in vivo* and evaluate their adequacy as surrogates with reduced pathogenicity during cheesemaking. With a genome engineering approach we generated three mutant strains in which genes *stx1* and *stx2* were substituted with antibiotic resistance cassettes to create UC4176(*Δstx1::kan*, Kan^R^), UC4177(*Δstx2::cat*, Cm^R^) and UC4178(*Δstx1::kan Δstx2::cat*, Kan^R^ Cm^R^), as confirmed by Sanger sequencing. Previous studies have deleted both *stx1* and *stx2* from STEC O157:H7 (Yokoyama et al., 2001; Ma et al., 2011), without evaluating the pathogenicity *in vivo*. We focused the first part of this study to assess the role of Shiga toxins in UC4224 and respective mutants *in vivo.* Our results showed that, when considering the deletion of either or both the *stx* genes, all three mutants presented differences in the lethality against *G. mellonella* larvae when compared to the parental strain. In particular, we observed that the double mutant UC4178 *Δstx1 Δstx2* showed highly reduced virulence with an increased LD_50_ of 2 Log dose when compared to UC4224 which shows a LD_50_ of 6 CFU/10 μL. Our study indicates that the presence of both *stx1* and *stx2* genes have a combined effect on the pathogenicity of STEC, in fact the single mutants UC4176 *Δstx1* and UC4177 *Δstx2* showed a lower virulence (1 Log increase of median lethal dose) than UC4224. No differences between strains producing Stx1or Stx2 toxins were detected in the *G. mellonella* model, differently from what was observed in other animal models (Xue et al., 2011). In line with our results, a previous study has shown that non-pathogenic *E. coli* strains are non-lethal to *G. mellonella* with inoculations of up to 10^7^ CFU/larvae (Zuppi et al., 2020). Our results highlight that the deletion of either or both the *stx* genes does not completely suppress UC4224 virulence, leading to suppose the involvement of LAA PAI, plasmid-encoded VFs, non-*stx* prophage encoded VFs, non-LEE T3SS effectors and other colonization contributing factors in delivering pathogenicity to the host (Cundon et al., 2018; da Campos et al., 2019; Vélez et al., 2020b; Cortimiglia et al., 2021; Sánchez et al., 2021). Indeed, previously, another study observed that the deletion of *stx* genes in the presence of other virulence factors reduces the pathogenicity. In this work, Habets et al. (2022) showed that non-STEC EPEC O80:H26 *E. coli* strains which correctly transduced with the Stx2d-phage, increased lethality in *G. mellonella* larvae, proving that the Stx2-phage confers partial virulence to a strain harboring other virulence factors (Habets et al., 2022).

After establishing the mutants as suitable substitutes with diminished virulence, the second part of our study focused on the evaluation of the phenotypic differences between the mutants and the parental strain. The three mutants and the parental strain were submitted to a pilot scale raw milk cheese production to assess their survival in the cheese matrix, which is typically subjected to different stressing conditions like temperature, pH, a_w_ and redox potential changes. The possibility to use less virulent strains to study how it reacts within cheese manufacturing is important in challenge tests to avoid using hazardous pathogens. The intrinsic attributes of cheese, related to the different production and ripening processes, should act as a barrier to bacterial growth. Along with this, the intrinsic microbiota of raw milk together with the starter cultures are expected to outcompete pathogens by lowering the pH (Baylis, 2009). Nevertheless, raw milk cheeses of different varieties (soft and semi-hard) have been described as sources of contamination or outbreaks of STEC, since they do not undergo pasteurisation and the production process is not effective in counteracting the proliferation of these bacteria (Schlesser et al., 2006; Caro and García-Armesto, 2007; Miszczycha et al., 2013, 2016; Peng et al., 2013; Ahmed and Samer, 2017; Celikl et al., 2021). However, STEC have been isolated from pasteurised milk cheese as well, possibly due to cross-contamination (Fereydouni and Darbouy, 2015; Callon et al., 2016; Cardoso and Marin, 2016). It has been demonstrated that the survival capacity of STEC in the cheesemaking environment is due to the activation of stress response systems (dos Santos Rosario et al., 2021). This mechanism includes the induction of sigma factor encoded by gene *rpoS,* as a response reaction to acid stress and can also be influenced by high pressure, cold, heat, UV radiation, H_2_O_2_ and the concentration of salt (Cheville et al., 1996; Robey et al., 2001; Mei et al., 2015; Li et al., 2018). Other SOS response regulons were identified in UC4224 that act together with the induction of σ^S^ such as *gadE,* coding for one of the most efficient acid stress regulators (Vanaja et al., 2009), osmotic regulator *ompR* and oxidative stress coping gene *katG* (dos Santos Rosario et al., 2021). In line with other studies (Dineen et al., 1998), our results showed that the acidity values found in dairy products do not limit the growth of UC4224 and its three mutants being able to grow at pH 4.5, a value substantially lower than that of cheese. In a previous work by Cheng et al. (2002), where *E. coli* O157 was treated with pH 5.5 for 4 and 5 h, resulting in higher resistance to 10% NaCl and a temperature of 55°C (Cheng et al., 2002). Another study has shown that certain strains of the O157 serogroup are able to survive at low pH between 3 and 4, although the ideal condition for their growth is at pH 7 (Meira et al., 2017). The presence of these survival mechanisms in STEC explains the fact that they can be isolated from different types of dairy products and dairy-related environments. In effect, the phylogenomic analysis conducted in this study elucidated that the distribution of the *stx* genes did not follow a particular pattern in relation to the isolation sources or serogroups. The most abundant were O6, O26, O157 and O174. The latter was also found in other semi-hard raw milk cheeses as reported in previous study (Cortimiglia et al., 2021). Other studies have stated that O174 strains were sporadically isolated during outbreaks yet they represented the most frequently isolated STEC isolates from cattle and foods (Stephan et al., 2008; Lorenz et al., 2013; Cundon et al., 2018). Our results indicated a similar behaviour of the parental strains and engineered strains, demonstrating that the genomic modification did not affect the possibility to use them to study various metabolic features useful in the cheesemaking process.

For the first time, we investigated the pathogenicity of O174:H2 non-LEE STEC highlighting that the virulence is related not only to *stx* genes but to other virulence factors. For this reason, further efforts should be done to gain a deeper knowledge on STEC from food regarding the importance of non-*stx* non-LEE virulence markers in defining the pathogenicity potential of dairy isolates. This work led to the creation adequate surrogates with decreased virulence for studies during food processing. In order to enhance the suitability and safety of these strains, further experiments need to be conducted to eliminate non*-stx* virulence factors.

## Data availability statement

The datasets presented in this study can be found in online repositories. The names of the repository/repositories and accession number(s) can be found in the article/Supplementary material.

## Author contributions

GM: methodology, investigation, and writing-original draft preparation. MB: formal analysis, investigation, and writing-original draft preparation. CC and DB: writing-review and editing. PC: conceptualisation, writing-review and editing, validation, visualisation, supervision, and project administration. All authors contributed to the article and approved the submitted version.

## Funding

This work was supported by the National Recovery and Resilience Plan (NRRP), Mission 4 Component 2 Investment 1.3 – Call for tender No. 341 of 15 March 2022 of Italian Ministry of University and Research funded by the European Union – NextGenerationEU. Award Number: Project code PE00000003, Concession Decree No. 1550 of 11 October 2022 adopted by the Italian Ministry of University and Research, CUP D93C22000890001, Project title “ON Foods – Research and innovation network on food and nutrition Sustainability, Safety and Security – Working ON Foods.”

## Conflict of interest

The authors declare that the research was conducted in the absence of any commercial or financial relationships that could be construed as a potential conflict of interest.

## Publisher’s note

All claims expressed in this article are solely those of the authors and do not necessarily represent those of their affiliated organizations, or those of the publisher, the editors and the reviewers. Any product that may be evaluated in this article, or claim that may be made by its manufacturer, is not guaranteed or endorsed by the publisher.
